# New Ideas for the Prevention and Treatment of Preeclampsia and Their Molecular Inspirations

**DOI:** 10.3390/ijms241512100

**Published:** 2023-07-28

**Authors:** Agata Sakowicz, Michalina Bralewska, Magda Rybak-Krzyszkowska, Mariusz Grzesiak, Tadeusz Pietrucha

**Affiliations:** 1 Department of Medical Biotechnology, Medical University of Lodz, Zeligowskiego 7/9, 90-752 Lodz, Poland; michalina.bralewska@umed.lodz.pl (M.B.); tadeusz.pietrucha@umed.lodz.pl (T.P.); 2 Department of Obstetrics and Perinatology, University Hospital in Krakow, 31-501 Krakow, Poland; magda@hi-gen.pl; 3 Department of Perinatology, Obstetrics and Gynecology, Polish Mother’s Memorial Hospital-Research Institute in Lodz, 93-338 Lodz, Poland; mariusz.grzesiak@gmail.com; 4 Department of Gynecology and Obstetrics, Medical University of Lodz, 93-338 Lodz, Poland

**Keywords:** antioxidants, antithrombin III, anti-inflammatory agents, apheresis, metformin, nitric oxide, nuclear factor kappa B, peptides, polyphenols, preeclampsia, probiotics, proton pump inhibitors, short interfering RNA, soluble fms-like tyrosine kinase 1, statins, vitamins

## Abstract

Preeclampsia (PE) is a pregnancy-specific disorder affecting 4–10% of all expectant women. It greatly increases the risk of maternal and foetal death. Although the main symptoms generally appear after week 20 of gestation, scientific studies indicate that the mechanism underpinning PE is initiated at the beginning of gestation. It is known that the pathomechanism of preeclampsia is strongly related to inflammation and oxidative stress, which influence placentation and provoke endothelial dysfunction in the mother. However, as of yet, no “key players” regulating all these processes have been discovered. This might be why current therapeutic strategies intended for prevention or treatment are not fully effective, and the only effective method to stop the disease is the premature induction of delivery, mostly by caesarean section. Therefore, there is a need for further research into new pharmacological strategies for the treatment and prevention of preeclampsia. This review presents new preventive methods and therapies for PE not yet recommended by obstetrical and gynaecological societies. As many of these therapies are in preclinical studies or under evaluation in clinical trials, this paper reports the molecular targets of the tested agents or methods.

## 1. Introduction

The second most common disease associated with the gestation period, recognised in 4–10% of all pregnant women, is preeclampsia (PE) [1]. It generally manifests as the sudden occurrence of high blood pressure (>140/90 mmHg) accompanied by proteinuria developing after week twenty of gestation. However, proteinuria is not required to indicate preeclampsia; it is sufficient for hypertension to be present in previously normotensive women, complicated by at least one of the symptoms indicating an organ dysfunction. Although obstetrical and gynaecological societies share the common view that the appearance of organ dysfunction should be included in the diagnostic criteria of PE, individual organisations recommend different definitions of organ dysfunction. More specifically, renal dysfunction can be indicated by serum creatinine concentration >1 mg/dL or >1.1 mg/dL; liver dysfunction by transaminase levels elevated twofold over the reference range or ≥40 IU/L; haematological disorders by blood platelet levels <100,000/µL or <150,000/µL; or haemolysis. In addition, neurological disorders indicated by cerebral stroke, altered mental status, visual symptoms or severe headache with hyperreflexia, or other clinical symptoms such as uteroplacental dysfunction or pulmonary oedema are included in diagnostics criteria [2]. In addition, preeclampsia is also associated with certain risk factors that differ between cases with the early-onset form, i.e., before week 34 of gestation, and the late-onset occurring after week 34 of gestation [3]. Among these risk factors, those associated with inflammation are also common, i.e., chronic hypertension, diabetes mellitus, renal disease, autoimmunological disorders, or obesity [4]. 

Although the phenomenon of preeclampsia has long been described in the literature, the pathomechanism remains unclear, and the only method known to halt the disease is premature induction of delivery, generally by Caesarean section. Therefore, there is a need for further research into new pharmacological strategies for the treatment and prevention of preeclampsia.

This article reviews the current state of knowledge about therapies that have been proposed for the prevention and treatment of preeclampsia but have not yet been officially recommended by obstetrics and gynaecological societies. As a lot of these therapies are still under investigation in preclinical studies, this review presents the results obtained from in vitro and in vivo models and indicates their possible modes of action. Therapies under investigation in phase one, two, or three clinical studies are also presented in this article. Although these clinical trial results have not been made public yet, the molecular pathways influenced by these drugs are discussed.

## 2. The Possible Pathomechanism of Preeclampsia

It is believed that preeclampsia is mainly caused by the improper formation of the placenta at the beginning of pregnancy, i.e., several weeks before the manifestation of the first clinical symptoms. 

Each gestation begins with an inflammatory reaction that develops in the maternal uterus before fertilisation. An increase in the activation of the nuclear factor kappa B (NFĸB) transcription factor in endometrial cells is already observed at the proliferative phase of the menstrual cycle [5]. This factor is known to regulate the expression of more than 400 genes including those related to inflammation (e.g., tumour necrosis factor alpha (TNFα), Interleukin 1, 6, 8 (IL1, IL6, IL8), or cyclooxygenase 2 (COX2)), antigen presentation (e.g., complement components B, C3, or C4), extracellular matrix degradation (e.g., metalloproteinases MMP1, MMP3, or MMP9), angiogenesis or apoptosis (e.g., p53, or proapoptotic Bcl-2 homologue (Bax)) [6,7,8,9]. Therefore, it is believed that NFĸB might play a critical role in preparing maternal tissues for the implantation, invasion of trophoblastic cells into the maternal decidua, and the transformation of maternal spiral arteries into wide and low-resistance vessels. However, in PE, the excessive activation of NFĸB augments the inflammatory reaction in the uterus; this disturbs the communication at the maternal–foetal interface, thus preventing correct placentation and maternal vessel remodelling. The levels of free radicals, inflammatory factors, including TNFα and IL6, as well as thromboxane 2, a product of arachidonic acid (AA) degradation by cyclooxygenases (COX), increase in maternal blood [10,11]. Moreover, the serum of preeclamptic mothers exhibits strong activation of the complement system, manifested as elevated C3a and C5a factors [12]. Under inflammatory conditions, the complement components, i.e., C1q, C4d, and the receptor for C5a, are diffused across the preeclamptic placentas [13,14,15,16,17]. Additionally, the incorrect remodelling of uterine vessels starves the placental cells of nutrients and oxygen, and so the cells start to secrete placental factors such as soluble fms-like tyrosine kinase 1 (sFlt-1) or soluble endoglin (sEng) into the maternal circulation [18]. These factors slow the process of angiogenesis and augment systemic inflammation and exacerbate maternal endothelium dysfunction. Moreover, sFlt-1 has been found to reduce the bioavailability of vasodilatory nitric oxide (NO) and increase the sensitivity of the endothelial cells to proinflammatory factors present in the maternal circulation [19,20]. These dysfunctions interfere with the mechanisms regulating blood pressure, exacerbating maternal hypertension and thus preeclampsia. 

## 3. The Preventive and Therapeutic Strategies Recommended by Obstetricians and Gynaecological Societies

Among the drugs recommended by obstetrical and gynaecological societies, aspirin (ASA) seems to be the most efficient for preventing preeclampsia, with data indicating around a 60% reduction in risk [21]. However, this drug is recommended only for high-risk groups, and its effectiveness depends on the time of starting therapy. Indeed, supplementation must begin before week sixteen of gestation; after this time, it has a less positive influence on gestation or can even increase the risk of an adverse outcome [21,22,23]. 

It is currently a mystery why starting aspirin supplementation after week sixteen is ineffective; it is also unknown which molecular pathways involved in the pathomechanism of PE are controlled by this agent.

ASA is mostly known as an inhibitor of arachidonic acid (AA) metabolism. It irreversibly blocks the activity of cyclooxygenase 1 and 2 (COX1, COX2) by acetylation, thus preventing the degradation of AA into prostaglandins (e.g., thromboxane and prostacyclin) [24]. Interestingly, some studies indicate that supplementation with low-dose ASA during pregnancy selectively blocks the production of proinflammatory thromboxane but not prostacyclin (PGI_2_), which is known for its vasodilatory action [25]. This anti-inflammatory activity may be supported by its ability to inhibit NFĸB activation and nuclear translocation in maternal and placental cells. ASA also influences the phosphorylation and further degradation of IĸBα, one of the most common inhibitors of nuclear factor kappa B [26,27]. It is possible that these mechanisms may be of particular importance at the beginning of a preeclamptic gestation, in which the excessive inflammatory reaction might disturb the cross-talk between the maternal and foetal sides. However, when the process of placentation is completed, and other pathways start to dominate in maternal and trophoblastic cells, ASA might be not sufficient to reduce the inflammatory reaction. 

Although it is able to regulate vasoconstriction by activating endothelial synthase of nitric oxide (eNOS), and inhibit apoptosis by acetylating p53 protein, it is not able to counter the problems associated with preeclamptic gestation [28,29]. As such, the initiation of ASA treatment after week 16 of pregnancy is ineffective. 

Although various gynaecological and obstetrical societies advise the use of ASA as a prophylactic for PE, the recommended dosage in not consistent. The World Health Organisation (WHO) advises a minimal dose of 75 mg per day for high-risk women whereas other societies such as the International Society for the Study of Hypertension in Pregnancy (ISSHP) recommends higher doses, even up to 162 mg/day; similarly, the week of gestation reserved for initiation of treatment ranges from 11 to 20, with the optimum below week 16 of gestation [27]. 

Similarly, the guidelines provided by obstetrical and gynaecological societies for treatment of high blood pressure in PE vary slightly (Table 1).

Although all drugs used to treat preeclampsia are believed to counter high blood pressure, i.e., the main symptom of preeclampsia, their mechanism of action extends much further. 

One of the most common antihypertensive drugs used to treat of preeclampsia is methyldopa. It acts as an agonist of α2-adrenergic receptors localised in the presynaptic space and inhibits the sympathetic system by controlling the release of norepinephrine from neurons. This medication substitutes for dihydroxyphenylalanine in the biosynthesis of neurotransmitters such as dopamine, norepinephrine, or epinephrine, and influences the production of their inactive forms. This impairs the signalling pathway from the baroreceptors, thus altering blood pressure [37]. Some studies indicate that methyldopa is implicated in the modulation of intracellular messengers including cyclic adenosine monophosphate (cAMP), known as a stronger inducer of Flt1 expression [38]. This might explain why methyldopa treatment inhibits the secretion of sFlt1 by the endothelial and placental cells after initiation, as observed in case–control and in vitro studies [38,39,40,41]. 

Interestingly, methyldopa also exerted a positive influence on endothelial cells by significantly increasing vascular endothelial growth factor (VEGF) production in the cell culture medium [39]. However, this effect was not observed in pregnant women; in fact, preeclamptic patients treated with methyldopa demonstrated significantly lower VEGF levels after 48 h from supplementation compared to the period before the initiation of treatment. The decrease in VEGF concentration was also dependent on the dose of methyldopa [42]. 

Another common drug used for reduction of high blood pressure in pregnancy is hydralazine, which was found to reduce VEGF levels in human umbilical vein endothelial cell (HUVEC) culture, thus negatively influencing the process of angiogenesis, migration, and proliferation in culture cells [43]. This mode of action was not observed in vivo. The intravenous infusion of hydralazine into mice significantly improved drug-mediated vasodilation, vascular tone, blood flow, and tissue perfusion. This rapid reaction occurred together with the acute induction of hypoxia factor type 1 alpha (HIF-1α) in the organs of the treated mice. In addition, the elevation of HIF-1α was accompanied by an increase in VEGF levels in both the serum and tissues isolated from animals [44]. It is therefore possible that in addition to the direct influx of the drug on the relaxation of arterial smooth muscle, the improvement in blood pressure parameters observed in preeclamptic women after hydralazine infusion may be due to the regulation of intracellular HIF-1α-dependent pathways, including those associated with VEGF production. 

Like methyldopa, hydralazine is also implicated in the regulation of sFlt1: it appears to reduce the production of sFlt1 and significantly increase the integration of trophoblastic cells (i.e., HTR8/SVneo cells) into the endothelial cells in preeclamptic environment (i.e., rich in TNFα factor) [39,45]. Additionally, hydralazine also appears to regulate the inflammatory reaction; high doses reduce the production of IL6 and TNFα in placental explants as well as in peripheral blood mononuclear cells (PBMC) [46,47]. Low doses stimulate IL10 production in PBMCs, which is important for correct gestation, and is depleted in preeclamptic pregnancies [46,47]. Moreover, studies on animal models indicate that hydralazine improves neovasculargenesis and inhibits the production of reactive oxygen species (ROS) in endothelial cells [48]. 

Nifedipine, the next most common agent recommended by obstetrical and gynaecological societies as the first-line drug for treatment of preeclampsia also manifests a number of activities that go beyond the regulation of blood pressure. Similar to hydralazine, this agent is believed to reduce ROS levels and inhibit the expression of various compounds, including metalloproteinase 13 (MMP13), IL1β, IL6, TNFα, and cyclooxygenase 2 (COX-2) [49]. Many of these factors have been implicated in the pathomechanism of preeclampsia, and all of them are under the control of NFĸB. Indeed, some studies suggest that nifedipine realises its anti-inflammatory functions via the regulation of the NFĸB activation pathway [50,51]. It has been suggested that the drug inhibits the phosphorylation of inhibitor of nuclear factor kappa B (i.e., IĸBα), thus preventing the translocation of NFĸB from the cytoplasm to the nucleus. When present at low levels in the nucleus, it is not possible for NFĸB to regulate its target genes [27,52]. This may also explain why nifedipine supplementation appears to reduce the expression of the adhesion molecules, i.e., intracellular adhesion molecules 1 (ICAM-1) or selectin E (SelE) [53]. Both particles are under the control of NFĸB and both are significantly exposed on the endothelial cells in the conditions of high levels in inflammatory (e.g., TNFα or IL6) and placental factors (e.g., sFlt1 or sENG), as well as “placental debris”, i.e., in the environment typical for preeclampsia [27]. Moreover, hydralazine treatment benefits patients with hypertension by ameliorating angiogenesis and increasing the bioavailability of nitric oxide [54,55,56]. 

Another drug recommended by obstetrician and gynaecological societies for first-line treatment of both mild (i.e., >140/90 mmHg) and severe (i.e., >160/110 mmHg) hypertension is the β-blocker labetalol [57]. This may also increase the accessibility of nitric oxide to the endothelial cells. An in vitro study found this drug to counteract inhibition of eNOS expression by TNFα, at the mRNA level, in human uterine microvascular endothelial cells [41]. Moreover, labetalol inhibits superoxide production: labetalol reduced ROS levels in both human and rabbits three hours from injection [58]. Other beta-blockers accepted for the treatment of pregnancy hypertension, e.g., esmolol, also demonstrate antioxidant activity by inhibiting superoxide generation [59,60]. Moreover some beta blockers influence the arachidonic acid metabolism, reducing platelet aggregation and thromboxane generation; both processes are linked to the progression of preeclampsia [60,61,62].

The drugs recommended by obstetrical and gynaecological societies for preeclampsia treatment are known to present broad mechanisms of action, i.e., reducing high blood pressure and improving inflammation and oxidative stress, and ameliorating both maternal endothelial cell and placental dysfunction; however, it remains unclear why the therapy confers no clear benefits to pregnant women, and why only “cure” for preeclampsia is delivery, often prematurely [63]. It is highly probable that the time of application with regard to week of gestation makes a difference; also, the period of exposure to the antihypertensive drugs may be too short to activate their additional mechanisms of action to benefit the patient. Indeed, the clinical symptoms of preeclampsia occur suddenly when the maternal compensative mechanism fails. At this stage of the disease, serious structural and metabolic changes in placental and maternal endothelial cells have already occurred. The maternal storm of cytokines and ROS is too great to be inhibited by drugs that possess only mild anti-inflammatory or antioxidant properties as side effects: the concentrations of the placental factors, e.g., sFlt1 and sENG in the maternal bloodstream are too high for them to be scavenged or neutralised by antihypertensive agents alone. As a result, the destruction of maternal vascular endothelial and glomerular filtration barriers continues, exacerbating the symptoms of disease and threatening maternal and foetal life. 

Therefore, there is a need to identify new preventive agents and drugs precisely focused on elimination at least one factor provoking the development or progress of preeclampsia. The mechanism of action of these drugs should not only focus on countering the factors impairing the metabolism of endothelial cells, but also treating, or preventing, the pathological mechanisms localised in the endothelial and placental cells. They should also aim to ameliorate the systemic inflammation and oxidative stress experienced by pregnant preeclamptic women.

## 4. Novel Therapies for the Prevention and Treatment of Preeclampsia

### 4.1. Statins—The Old Friends of the Cardiovascular System Offering New Perspectives in Preventing Preeclampsia

Statins inhibit Hydroxymethylglutaryl coenzyme A (HMG-CoA) reductase; an enzyme implicated in the biosynthesis of cholesterol in liver cells. Following oral administration, statins are rapidly absorbed and linked to the serum protein, and then their further metabolism depends on their physical properties, i.e., hydrophilic statins require active transport whereas lipophilic agents undergo passive diffusion through the cellular membrane of liver cells. They downregulate the level of intrahepatic cholesterol, improving the systemic expression of receptors for low-density lipoproteins (LDL), thus lowering the level of lipids in the bloodstream [64]. However, statins offer other clinical benefits to patients than just reducing lipoprotein levels. 

Statins are implicated in the regulation of inflammation, whose exacerbation is believed to play a role in preeclampsia development. The drugs lower the levels of C-reactive protein, and this mode of action is independent of their ability to inhibit HMG-CoA reductase activity [64]. In vitro studies indicate that statins may block the secretion of proinflammatory cytokines and prevent to the differentiation of T lymphocytes into the Th1 subfraction, as observed in cells exposed to lipopolysaccharide (LPS), i.e., a strong activator of NFĸB [65]. Indeed, statins can inhibit NFĸB activation by different mechanisms, either dependent on or independent of lipid metabolism. 

Statin treatment led to a reduction in LDL level, resulting in a depletion of their oxidative forms (oxy-LDL), these being strong stimulators of Toll-like receptors (TLRs); TLR signalling is one of the most important mechanisms that elevate IL6 and TNFα levels following increased NFĸB transcriptional activity [66]. Statins were also known to block the phosphorylation of nuclear factor of kappa light polypeptide gene enhancer in B-cells inhibitor alpha (IĸBα) in the proteasome, thus resulting in its further degradation. The inactive form of IĸBα remains linked to the NFĸB in the cytoplasm, thus making the NFĸB unable to migrate into the nucleus, where it regulates gene expression via its binding motif element on DNA [67]. Additionally, statins inhibit the mechanism of NFĸB activation by blocking phosphatidylinositol 3-kinase (PI3k)/kinase protein B (Akt) signalling. This pathway is activated in endothelial cells in response to TNFα stimulation [68]. It has also been Implicated in the generation of ROSIch, Iike inflammatory factors, have been involved in the pathomechanism of preeclampsia [69,70]. 

There is growing evidence that statin therapy influences the secretion of sFlt1 and sENG particles from primary trophoblast and placental cells living under unfavourable conditions [71,72]. This action has been observed both in vitro and in vivo. Indeed, studies based on various mouse and rat preeclamptic models indicate that animals treated by pravastatin demonstrated a depletion in sFlt1 and/or sENG factors [73,74,75,76]. This mechanisms might be dependent on the HMG-CoA reductase pathway [77] or it might be connected to the ability of statins to elevate haem oxygenase 1 (HO1) level, which is suppressed in the blood of preeclamptic mothers [78]. Both sFlt1 and sENG act as the soluble forms of receptors for PlGF, VEGF, or TGFβ in the bloodstream, thus preventing them from binding to the receptors localised on the cells [79] and disturbing the cellular processes regulated by PlGF, VEGF, or TGFβ. This results in endothelial damage, inhibits angiogenesis and impairs the process of vasodilatation [72]. Indeed, the endothelial damage is linked to the downregulation of endothelial nitric oxide synthase (eNOS) production, leading to a reduction in nitric oxide (NO) level, increased vasoconstriction and the development of hypertension.

Surprisingly, statins exert a beneficial influence on the endothelial cells, even in advances stages of dysfunction. They most likely upregulate the expression of eNOS by the PI3k/Akt mechanism or by the upregulation of haem oxygenase 1 level; this most likely facilitates their ability to elevate NO level and vessel relaxation [80,81]. Numerous studies have reported statin, especially pravastatin, to have a positive influence on blood pressure and a reduction in the risk of adverse pregnancy outcome (Table 2). At present (June 2023), a few studies evaluating the use of statins (i.e., pravastatin, rosuvastatin and atorvastatin) in the preeclamptic population are registered in the clinicaltrials.gov database, and three of them are still in progress (i.e., NCT04303806, NCT01717586, NCT03944512).

Although statins have been found to have a positive influence on reducing the risk of preeclampsia, or stabilizing its clinical features, the American Food and Drug Administration (FDA) still does not recommend the use of these drugs for all pregnant women. Statins reduce cholesterol levels, depleting its accessibility to the developing foetus and thus elevating the risk of miscarriages or foetal congenital defects. Indeed, supplementation of pregnant animals with the supraphysiologic doses of statins has been found to have a teratogenic effect on the foetuses [87]. Additionally, statin treatment has been found to impair gestational outcomes [88,89]. However, these initial concerns were not confirmed in recent animal and clinical trials, and in July 2021, the FDA allowed the use of statins during pregnancy but only in certain cases: among women with familiar hypercholesterolemia, those suffering from severed LDL cholesterol level, those with cardiovascular diseases, or in cases where the benefits are judged to outweigh any risk [90].

### 4.2. Anti-Inflammatory Agents in the Treatment of Preeclampsia

Placental cells expose the proteins CD55 and CD59 on their surface-inhibiting complement factors to regulate maternal immunological tolerance to the developing semi-allogenic foetus [91]. The strength of this exposure seems to be inflammation dependent. Preeclamptic placentas exhibit twofold and fourfold upregulation of genes coding for CD55 and CD59, respectively, suggesting that placental cells may attempt to compensate for the excessive reactivity of the maternal complement system [15], characterised by elevated C3a and C5a components in preeclamptic maternal plasma [12]. 

Additionally, immunohistochemical studies indicate that preeclamptic placentas demonstrate elevated deposition of the C4d complement and the receptor for the C5a complement (C5aR) [14,15]. The activation of the placental C5a/C5aR pathway results in placental dysfunction; in vitro studies indicate that complement C5a can inhibit angiogenesis and trophoblastic cell migration (i.e., HTR8/SVneo) [14]. Similarly, studies on tissues obtained from rats and mouse models of preeclampsia confirm strong activation of the complement system in preeclampsia, with increased expression of C3 complement factor observed in the vessels and placentas. Moreover, in mice, augmented generation of C5a complement factor was associated with impaired angiogenesis and VEGF production, and overexpression of sFlt1 by placental cells [92]. These findings suggest that the blockade of the complement system, leading to the inhibition of inflammation, might be an effective method for mitigating the symptoms of preeclampsia. 

Eculizumab (Soliris) is a humanised monoclonal antibody class (Ig)G2/4 kappa that binds to the C5 complement. It inhibits the cleavage of C5 into its active forms, i.e., C5a and C5b, and hence it is believed that it might help the preeclamptic mother and foetus by silencing inflammation. Generally, this drug offers benefits for patients suffering from paroxysmal nocturnal haemoglobinuria, atypical haemolytic uremic syndrome, and other autoimmunological diseases that can lead to PE, such as antiphospholipid syndrome or lupus erythematosus: dysregulation of the complement system is a typical sign of PE [93,94,95,96,97]. 

Although it is well established that complement system is dysregulated in preeclampsia, little information exists about the beneficial effect of use of this drug in pregnant women. Some studies indicate that this drug is well tolerated and has a low potential to cross the placental barrier or to appear in maternal milk [98]. While eculizumab is generally not detected in the new-borns of mothers treated during pregnancy [99], supplementation of pregnant animals by two-to-eight times the standard dose of an analogous drug (Soliris) resulted in in an increased rate of developmental abnormities or foetal death; as such, the FDA awarded the drug a category C for pregnancy, i.e., it is allowed for use in pregnancy if the potential benefit justifies the potential risk to the foetus [100]. 

Therefore, the first reports concerning the use of eculizumab for preeclampsia treatment are based on cases suffering from other diseases coincident to preeclampsia, e.g., thrombotic microangiopathy (TMA) or antiphospholipid syndrome. These studies found that eculizumab can extend the time of gestation, which is especially important for women who develop early onset preeclampsia. Indeed, Soliris treatment extended gestation for another 17 days in women with TMA who developed the HELLP syndrome (i.e., H*—*haemolysis; EL*—*elevated liver enzymes level; LP*—*low platelets) at week 26 of gestation [101]. Eculizumab was found to have similar effects in other cases presenting preeclamptic symptoms between weeks 27 and 28 of gestation and additionally suffering from TMA or antiphospholytic syndrome [102]. At present, Soliris is in the second clinical trial phase (NCT04725812) to confirm its ability prolong pregnancy complicated between weeks 23 and 30 by preeclampsia. 

Another candidate drug for the prevention of preeclampsia is etanercept, a TNFα inhibitor consisting of a complex of the extracellular fragment of the TNFα receptor with the Fc fragment of the human IgG antibody; this has offered promise in in vitro studies and animal preeclampsia-like models. Preeclampsia is strongly linked to the elevation of TNFα in maternal plasma. The administration of TNFα to pregnant animals increases the levels of placental factors, e.g., sFlt1 or sENG, in the blood and stimulates the maternal organism to produce autoantibodies against angiotensin II type I receptors (AT1-AA), which promotes placental insufficiency, renal damage, and maternal endothelial dysfunction [103,104]. The blockade of TNFα by etanercept in a stroke-prone spontaneously hypertensive rat (SPHSR) model improved the maternal blood pressure and function of the uterine artery, thus improving pregnancy [105]. 

Etanercept was also found to positively regulate blood pressure in a RUPP rat model of preeclampsia and additionally improve natural killer cell activation in maternal blood and placental samples [106]. Administration of etanercept also lowered the levels of circulating sFlt1 and ROS in placentas in an RUPP rat model of PE [107,108]. 

However, both animal and human studies indicate that this agent undergoes transplacental transmission and thus may influence foetal development [109,110]. Indeed, both the placentas and offspring of rats treated with etanercept presented some defects, i.e., the reduction in weight, visceral or skeletal abnormalities, and depletion of the area of the placental junctional zone in comparison to controls [111]. In humans, etanercept has been used to treat autoimmunological diseases even in gestation, resulting in significant decreases in placental–maternal ratio [110]; moreover, anti-TNFα therapy entailed a reduction in new-born weight and increased the risk of preterm bright and caesarean section [112]. 

The data regarding the influence of etanercept on blood pressure in mothers at high risk of preeclampsia suffering from autoimmunological diseases are also not consistent. Some randomised trials indicate that women treated with etanercept for immune diseases (a risk factor of PE) to the end of week 10 of gestation experience a lower risk of pregnancies complicated by hypertension than a placebo group [113]; however, other studies indicate no significant differences between exposed and nonexposed groups [114]. Additionally, some scientific reports suggest that therapy targeting TNFα might increase the risk of preeclampsia development in the population of treated women due to autoimmunological diseases [115,116]. However, none of these studies examined the influence of etanercept on the treatment of preeclampsia. Therefore, further clinical trials are warranted to determine whether inhibitors of TNFα might be use for the treatment or prolongation of gestation of women presenting the clinical symptoms of preeclampsia.

Sulfasalazine is an anti-inflammatory and antioxidant drug adopted for treatment of autoimmune bowel disease or rheumatoid arthritis. It is believed to be a potent inhibitor of NFĸB nuclear translocation. An in vitro study on SW620 human colonic epithelial cells previously stimulated by TNFα, i.e., a strong activator of NFĸB, found similar cytoplasmic NFĸB levels between cells treated with sulfasalazine and unstimulated controls. This may be the route by which the drug inhibits phosphorylation and thus degrades NFĸB inhibitors [117,118]. In consequence, the transcriptional activity of factor kappa B is suppressed, downregulating the expression of genes coding for inflammatory agents such as IL1β, IL6, IL8, or TNFα [119]. 

The ability of sulfasalazine to inhibit NFĸB activity, and thus the levels of inflammatory factors, make it a strong candidate for treating preeclampsia. This drug reduces high blood pressure in pregnant mice developing preeclampsia caused by injection of a nitric oxide synthase antagonist, i.e., L-NAME [120]. This suggests that sulfasalazine may prevent the endothelial dysfunction least by upregulating the activity of eNOS and the production of vasodilatory factors such as nitric oxide [121]. Sulfasalazine may exert its positive influence on vasoactivity of maternal vessels through its potential to increase placental PlGF production and reduce the secretion of sFlt1 in an epidermal growth factor receptor-dependent manner [122,123,124]. However, results of the early phase of clinical trials (ACTRN12617000226303) determining the pharmacokinetics of sulfasalazine and its effect on the clinical and biochemical parameters of PE are still under analysis.

Hydroxychloroquine (HCQ) is an immunomodulatory agent that can relieve inflammation. In addition to its antimalarial properties, it is frequently used for treating autoimmune diseases, including those that are risk factors for preeclampsia, i.e., lupus or antiphospholipid syndrome [125,126]. HCQ inhibits NFĸB activity by blockade of the phosphorylation of kappa B inhibitor, thus downregulating the levels of inflammatory factors controlled by NFĸB [127,128]. In consequence of hydroxychloroquine supplementation, the sFlt1 secretion is reduced in cytotrophoblastic cells, and proangiogenic factors, e.g., PlGF are increased in primary HUVECs [129]. Additionally, as HCQ possesses antithrombotic activity, it might prevent fibrin formation and thus eliminate the risk of placenta insufficiency and the development of preeclampsia [130,131].

Several studies indicate that HCQ is a promising agent against preeclampsia. Most clinical studies have included women with a high risk of preeclampsia who are also suffering from autoimmunological diseases. HCQ supplementation was associated with a higher rate of live births, a lower prevalence of pregnancy morbidity, and a lower chance of preeclampsia development [132,133,134]. However, little is known about the preventive effect of HCQ among women without autoimmunological diseases. This gap is currently being addressed by one ongoing study (NCT05287321, HUGS; Phase 3) registered in the clinicaltrials.gov database. 

### 4.3. Therapies Targeting sFtl1 and Its Signalling

When the access to oxygen and nutrients is restricted due to incorrect placentation and maternal spiral uterine vessel transformation, placental cells produce a soluble receptor called sFlt1. Production is predominately governed by three isoforms of mRNA: sFLT1-i13 short, sFLT1-i13 long, and sFLT1-e15a [135]. Among women developing preeclampsia after week 20 of gestation, high levels of sFlt1 can be observed in the blood as early as in the first trimester [136]. The receptor downregulates the process of placental angiogenesis and maternal systemic inflammation and disrupts the maternal endothelium. In one study, injection of exogenous sFlt1 into animals induced preeclampsia-like symptoms; however, the increase in blood pressure was independent of the sex and the pregnancy status of laboratory animals [137,138]. It appears that sFlt1 may induce the clinical symptoms of preeclampsia, and as such, it is possible that removing sFlt1 from maternal blood or inhibiting its action might resolve its negative influence and any symptoms of disease. At present, various experimental therapies are under consideration in clinical trials. 

#### 4.3.1. Apheresis—Scavenger of sFlt1

The most invasive therapy under consideration for treating preeclamptic women is therapeutic apheresis adapted for extracorporeal removal of sFlt1 from maternal blood. Although this is a new idea, pregnancy is not a contradiction to apheresis: the method was accepted by the American Society for Apheresis for treating expectant women suffering from autoimmunological disorders, red cell autoimmunisation diseases, or hypercholesterolemia [139,140]. The first experimental therapies were based on heparin-mediated extracorporeal low-density lipoprotein (H.E.L.P.) apheresis; surprisingly, the removal of lipoproteins by this method also resulted in that of other agents such as TNFα, sFlt1, E-Selectin, endothelin-1, lipopolysaccharide binding protein, and the positive proteins of acute phase of inflammation [141]. However, a later clinical trial (NCT01967355) identified H.E.L.P. apheresis as an ineffective method for removing sFlt1 from maternal circulation. Nevertheless, this therapy has proven to be safe and allowed to extend the duration of pregnancy in the case of preeclampsia [142]. 

The later modification of the apheresis column, and the device for this method, delivered new possibilities for eliminating sFlt1 from maternal circulation. Whole-blood apheresis using a negatively charged dextran sulphate cellulose (DSC) column significantly reduced the soluble form of Fms-like tyrosine kinase-1 from preeclamptic blood. The method also allowed for the reduction in proteinuria and blood pressure without apparent adverse event to mother and foetus. However, the whole procedure had to be repeated twice to prolong the pregnancy for the next 15–19 days or three times for another 23 days [143]. Similar results were observed by Nakakita et al. [144], who used DSC apheresis to rescue a mother and foetus from severe preeclampsia that unusually developed before week 15 of gestation. The whole procedure was repeated thirteen times, staring from week 19 and extending the gestation for the next four weeks [144]. 

To improve sFlt1 elimination from maternal blood, the whole-blood apheresis was modified by plasma apheresis using a special device that first isolates the maternal plasma and then passes it through a plasma-specific dextran sulphate (PSDS) column. Although this method eliminates up to 28% of sFlt1, reduces the protein/creatinine ratio by up to 44% and decreases blood pressure, a similar prolongation of pregnancy was achieved as for whole-blood DSC apheresis, i.e., eight days for women treated once and 15 days for cases treated multiple times [145]. These results were confirmed elsewhere [146]. 

However, these observations were contradicted by those of Phase II of the ADENA clinical trial (NCT02286284) testing dextran apheresis. The trial had to be interrupted due to side effects of apheresis, i.e., occurrence of secondary uncontrolled hypertension with blurred vision during the first week of treatment [147,148]. The utility of apheresis for removing sFlt from the maternal bloodstream is now being verified by a new study (NCT02923206) currently registered in the ClinicalTrials database. 

#### 4.3.2. Inhibition of Synthesis of sFlt1

Recent research has indicated another potential alternative therapy to apheresis, based on the inhibition of sFlt1 production using three mRNA particles: sFLT1-i13 short, sFLT1-i13 long, and sFLT1-e15a. All are upregulated in preeclamptic placentas, providing a common protein product, i.e., sFlt1, which is released in excess from placental cells into the maternal bloodstream. The production of sFlt1 in placental cells is managed by small interfering RNA (siRNA) particles; these can selectively silence the production of sFlt1 but not full-length Flt1, a complete receptor for proangiogenic factors localised on the placental and vascular cells [149]. Moreover, it is possible to modify the structure of these siRNAs to make them favour tissues with high blood flow, such as the placenta; such modifications also alter the stability of the siRNAs, but with no discernible toxicity [135,150]. Studies on pregnant animals indicate that cholesterol-conjugated siRNA (hsiRNA) can reduce sFlt1 levels by up to 50% in maternal mouse blood, and over 50% in pregnant baboons; in both cases, preeclampsia was induced by uteroplacental ischemia (UPI) caused by ligation of the single uterine artery. Moreover, at about two weeks after the injection of the cholesterol-conjugated siRNA, the blood pressure and the proteinuria of the UPI baboons were reduced in comparison to sham-operated controls [135]. 

The siRNA connected to a nanocarrier, i.e., polyamidoamine (PAMAM) reduced blood pressure in rats with preeclampsia obtained after TNFα injection; in addition, treatment significantly lowered the level of sFlt1 in blood and urine in comparison to control. Additionally, the treatment improved the weight both foetuses and placentas of preeclamptic rats [150]. The results of studies on animal models were so promising that this method of treatment is still in development. At present, a number of modified particles of siRNA targeting sFlt1 mRNA intended for treating preeclampsia are under patent protection, e.g., EP3277815B1 (expiry in 2036), US9925261B2 (expiry in 2023) or CN113116819A (expiry in 2041). In addition, the U.S. Food and Drug Administration (FDA) cleared an investigational new drug (CBP-4888) based on siRNA therapy to treat preeclampsia in 2023. The safety, tolerability, and pharmacokinetics of the single ascending dose of CBP-4888 will be evaluated in healthy, nonpregnant women in a clinical randomised, double-blind, placebo-controlled trial (NCT05881993; Phase 1).

#### 4.3.3. Therapeutic Peptides Interfering with sFlt1 Signalling

The idea of use of peptides, i.e., small well-ordered chains of amino acids with a molecular mass not exceeding 5000 Daltons, for treating diseases was conceived in the first half of the twentieth century. Peptides may act as inter alia hormones, growth factors, or neurotransmitters, and can inhibit factors whose overproduction is related to the development of disease. They are inexpensive to produce and offer high specificity and low immunogenicity. However, they require some modifications to improve their therapeutic potential, e.g., by increasing their membrane permeability and half-life after administration [151]. 

Elastin-like polypeptide (ELP) is a nonimmunogenic, engineered particle composed of a repeating five amino acid motif; it is used as a carrier to stabilise therapeutic peptides and to protect them against degradation and removal from the organism [151,152]. The administration of a chimera of ELP linked with the vascular endothelial growth factor isoform type A (VEGF-A) or isoform type B (VEGF-B) to rats with PE induced by surgery, relays on the reduction of uterine perfusion pressure, was found to significantly block averse pregnancy [153,154]. VEGF-B is a structural homologue of VEGF-A, and both bind to the VEFGR1 receptors, including their soluble version, i.e., sFlt1 [155]. Therefore, the therapeutic peptides, i.e., ELP-VEGF-A and ELP-VEGF-B, reduce the level of free sFlt1, restoring angiogenic balance and thus improving maternal blood pressure. Moreover, the administration of the chimera ELP-VEGF-A affected the nitric oxide level in the kidneys of pregnant rats; in fact, nitrate and nitrile were found to be excreted in the urine, indicating that the drug as a potent inducer of NO signalling [153]. Similar results were obtained for the chimera ELP with VEGF isoform type B; however, this therapeutic peptide seemed to be a weaker proangiogenic driver than ELP-VEGF-A. It demonstrates lower stimulation of endothelial cell proliferation and matrix invasion than complex carriers isoform A, as indicated by in vitro studies [154]. Interestingly, these therapeutic peptides are safe for foetuses; the data indicates that ELP is a drug carrier sequestered in the maternal system and although it might localise in the placenta, it is not detected in the circulation of foetuses [153,156].

Although the therapies using ELP for carrying therapeutic peptides seem to be promising, at present, they are not under clinical investigation.

### 4.4. Novel Therapies Targeting the Production of Nitric Oxide

The correct course of gestation is strongly related to the synthesis and bioavailability of nitric oxide (NO) for maternal and placental cells. Indeed, NO is critical for the regulation of maternal vascular tone and for adapting the maternal cardiovascular system to the physiological changes associated with pregnancy, and thus for placental and foetal development. Therefore, the level of NO increases significantly in the first trimester and remains high throughout a normal pregnancy, and for the next few weeks after postpartum [157,158]. In preeclampsia, this trend is disturbed, as NO levels might be more than four times lower than in an uncomplicated gestation [157]. This has been found to be associated with a depletion in L-arginine-to-asymmetric dimethylarginine (ADMA) ratio in maternal blood. This indicates that L-arginine, a precursor of NO, is efficiently inhibited by its compete inhibitor, i.e., ADMA [159]. Therefore, supplementing the blood of preeclamptic mothers with precursors of NO may improve blood pressure and block the clinical symptoms of preeclampsia. 

This opinion is confirmed by data obtained from animal models. L-arginine administration to pregnant rats presenting preeclampsia syndromes generated by L-NAME, an inhibitor of endothelial nitric oxide synthase (eNOS), significantly reversed the adverse outcome to pregnancy. Both blood pressure and the level of albumin in urine were diminished. Similar concentrations of placental antiangiogenic, i.e., sFlt1 factor, and proangiogenic factors, i.e., PlGF and VEGF, were observed between rats receiving L-NAME + L-arginine and controls that did not receive L-NAME. Moreover, supplementation with L-arginine restored the level of circulating eNOS to basal levels in PE rats, suggesting that it improves NO production in maternal endothelial cells [160]. L-arginine was also found to have beneficial effects against PE in randomised controlled trials. It was found to effectively reduce the risk of occurrence of disease in a high-risk population in one study (NCT00469846) [161], and to be a promising drug for reducing the high blood pressure during gestation in another [162,163]. At present, a precursor of L-arginine, L-citrulline, is under evaluation as a preventive agent against gestational hypertension (NCT04979793; Phase 1). It was found to improve the function of the maternal endothelium in animal models of preeclampsia by reducing blood pressure and to improve placentation and foetal growth in Dahl salt-sensitive rats [164,165]. 

## 5. Other Agents for the Prevention or Treatment of Preeclampsia Currently under Investigation

### 5.1. Proton Pump Inhibitors

Five independent studies investigating the adaptation of esomeprazole for the prevention or treatment of preeclampsia can currently be found in the *clinicaltrials.gov* database (June 2023). Four of these studies, viz. NCT03717740, NCT03724838, NCT03717701, NCT03213639, ended before the beginning of the second quarter of 2022; the fifth study, in Phase 1, is still opening. Like lansoprazole, omeprazole, pantoprazole, and rabeprazole, esomeprazole is a proton pump inhibitor (PPI), used to relieve symptomatic gastric acid reflux by decreasing acid secretion. These drugs have been included as category B, i.e., safe to use in gestation, by the U.S. Food and Drug Administration. 

However, the value of these drugs for the treatment or prevention of preeclampsia remains uncertain. Atwa et.al [166] found that esomeprazole can prolong gestation in a study on a population of 160 expectant mothers with preeclampsia, and that treatment results in fewer PE complications, e.g., haemorrhages or intrauterine foetal death, or neonatal invasive care resulting from hypertension in pregnancy [166]. However, daily supplementation of 40 mg esomeprazole between weeks 26–37 of gestation did not prolong the pregnancy in the South African population, as shown in the results of a double-blind randomised control study [167]. This study did not also note any significant differences in the level of sFlt1 in the blood of preterm preeclamptic women treated with esomeprazole and those that were not [167]. However, another study found proton pump inhibitors to regulate the levels of placental-delivered factors (i.e., sFlt1, sENG) in the blood of women suffering from gestational hypertension, preeclampsia, and HELLP syndrome. These drugs selectively reduced sFlt1 level, but, surprisingly, the concentration of PlGF, i.e., ligand for the soluble receptor of Flt1 was stable. Moreover, with the course of sFlt1 reduction, the levels of endoglin and endothelin 1 (ET1) were also diminished, and these parameters correlated positively with each other [168]. 

Similar observations were made by the functional studies on primary human tissues, i.e., trophoblast, placental extracts from preeclamptic pregnancies and endothelial cells, as well as on a mouse model of preeclampsia (where sFlt1 is overexpressed in placenta) [169]. It is believed that proton pump inhibitors upregulate the haem oxygenase 1 (HO1) level that attenuates placental ischemia-induced hypertension, partially through normalization of the sFlt1-to-VEGF ratio in the placenta [169,170]. Moreover, proton pump inhibitors are known for their antioxidant properties and they exert anti-inflammatory effects [171]. Indeed, some of the anti-reflux agents, e.g., lansoprazole, are implicated in the regulation of NFĸB activity and In the mechanism supressing the phosphorylation of IĸBα (an NFĸB inhibitor) and its proteasome degradation. Additionally, these agents inhibit the phosphorylation of extracellular signal-regulated kinase (ERK) and thus attenuate the pathway that enhances the proliferation and apoptosis of trophoblastic cells in preeclamptic placentas [172,173]. Numerous reports have also provided evidence that esomeprazole, lansoprazole, and omeprazole may positively regulate endothelial cell functions. The umbilical endothelial cells treated with IL1β present a high expression of adhesion molecules i.e., intercellular adhesion molecule-1 (ICAM-1) and vascular cell adhesion molecule-1 (VCAM-1); however, these effects were attenuated after proton pump inhibitor treatment. Similarly, these drugs reduced the adhesion of immunological cells, i.e., neutrophils to the endothelial cells under inflammatory conditions [174,175]. Although the positive influx of proton pump inhibitors on the function of endothelial cells has been emphasised in numerous studies, the influx of these drugs on the production of nitric oxide are divergent. Some studies indicate that proton pump inhibitors are confounders responsible for the elevation of asymmetric dimethylarginine (ADMA). ADMA, being strongly elevated in the maternal blood of preeclamptic mothers, inhibits endogenous nitric oxide synthase and thus reduces the generation of nitric oxide at the vascular cellular level [176,177]. Despite this, other studies indicate PPI treatment increases vasorelaxation by upregulating the phosphorylated, i.e., active, form of nitric oxide synthase. This effect was observed both a mouse model of preeclampsia and ex vivo, i.e., in whole maternal vessels from PE patients [169]. Moreover, PPI treatment elevates the level of chromogranin A (CgA), a precursor for peptides with vasodilative properties, such as catestatin [178]. Both agents are synthesised in placental cells, and have been proposed as new markers of preeclampsia [179,180].

### 5.2. Metformin

Metformin improves glucose metabolism, and hence might be safely used in pregnancy: it has been classed as category B by the FDA. It reduces the insulin resistance characterising diseases regarded as risk factors of preeclampsia such as diabetes or obesity. Moreover, metformin mitigates endothelial dysfunction via an NFĸB-dependent mechanism. Indeed, NFĸB inhibitor phosphorylation was found to be blocked in cells cultured in a glucose-rich environment after exposure to metformin [181]. This results in the downregulation of proteins controlled by NFĸB, such as the inflammatory factors TNFα and IL1β, and the adhesion factors intracellular adhesion molecule type 1 (ICAM1), vascular cell adhesion molecule type 1 (VCAM1), or selectin E; this was also observed for the HUVEC cultures after metformin treatment [182]. 

As metformin ameliorates the higher NFĸB activity, overexpression of inflammatory factors and adhesive proteins, and upregulation of sFlt1 and sENG that are characteristic of preeclampsia, it may well serve as an effective treatment strategy. The impact of metformin treatment between weeks 26 and 32 on the health of preeclamptic women was investigated in a clinical trial (PACTR201608001752102). However, although it was found to be beneficial for prolonging gestation, it did not influence the maternal levels of sFlt1 and sENG or new-born birthweight in comparison to a placebo group [183]. Interestingly, metformin only demonstrates positive effects against preeclampsia in pregnant women suffering from diabetes or obesity. In women developing gestational diabetes, metformin reduces the occurrence of symptoms of gestational hypertension or PE compared to population receiving both insulin and metformin [184]. In addition, a meta-analysis of randomised trials indicated a reduced risk of preeclampsia in the population of women receiving metformin but only compared to those treated with insulin [185]. A four-times lower likelihood of preeclampsia occurrence (OR = 0.24; 95%Cl 0.10–0.61; *p* = 0.001) was also observed in a study on obese (BMI > 35 kg/m2) patients without diabetes who received metformin from the beginning of the second trimester until delivery compared with the obese control group [186]. At present, the effect of metformin administration to pregnant women before week 12 of gestation on the occurrence of preeclampsia is under examination in two clinical investigations: NCT04855513 (known as the PREMED study) and NCT03570632 (study in Phase 4). Two studies examining the synergic effect of metformin with acetylsalicylic acid or with esomeprazole are registered in the clinicaltrials.gov database, one examining the ability to reduce the risk of preterm cases of preeclampsia (NCT05580523) and the other as treatment (NCT05232994; Phase 1).

### 5.3. Antithrombin III

Preeclampsia is also characterised by hypercoagulability, which may be one of the reasons for the low level of platelets in the blood of preeclamptic women. In addition, the excessive activation of the coagulation cascade results in the intravascular consumption of anticoagulant factors, including antithrombin III. This depletion in antithrombin III level may also result from its restricted synthesis by the liver, its excretion in the urine, its shift to the interstitial compartment, or increased protein catabolism [187]. Antithrombin deficiency enhances intravascular clotting formation, and thus fibrin deposition, for example in preeclamptic placentas [131]. 

Therefore, anticoagulation agents may play a role in the treatment of preeclampsia. It was found that daily intravenous injection of antithrombin prolonged preeclamptic gestation and improved the biophysical parameters of pregnant mothers with severe preeclampsia and their foetus [188,189]. However, this finding was contradicted by a recent double-blind, placebo-controlled trial performed across 23 hospitals (NCT02029135), which found that antithrombin treatment did not prolong gestation nor improve neonatal or maternal outcome among women with severe preeclampsia [190]. At present, the potential of antithrombin (known as KW-3357) for treatment of early-onset preeclampsia is under examination in a randomised, placebo-controlled, double blind study (NCT04182373; Phase 3).

### 5.4. Natural Products, and Their Power to Reduce the Risk of Preeclampsia

In expectant women, changes in the hormonal status and in maternal dietary patterns influence the composition of the gut, vaginal, and oral microbiota [191,192]. Such shifts, and thus changes in the metabolites produced by the constituent microorganisms, might influence the maternal metabolism and immune system, including the changes in the levels of Th1/Th17, i.e., proinflammatory cytokines [193,194]. Indeed, nonpregnant germ-free mice were found to demonstrate increased body weight, impaired glucose metabolism and greater inflammation after exposure to the maternal microbiome obtained from the third trimester of expectant women [195]. As maternal metabolic changes and disturbances in the balance between Th1/Th2 cytokine profile are strongly related to placental-associated diseases such as preeclampsia and intrauterine growth restriction or HELLP syndrome, it is possible that disruptions in the maternal microbiome may influence their development [196]. 

Although it seems obvious that the vaginal and placental microbiomes should play a significant role in the correct course of gestation, this has not been widely addressed in the literature. The nature of the placental microbiome remains unclear, as the placenta is considered as sterile and its colonisation by microorganisms is perceived as pathological [197]. For example, one study identified ten genera of bacteria, including those most commonly linked with gastrointestinal infection, e.g., *Salmonella* and *Escherichia*, or with respiratory tract infection or periodontitis in preeclamptic placentas, but not in normotensive women, which were negative (germ-free) [198]. Another study found the *Lactobacillus* genus, typical of the vaginal environment, to be the most prominent bacteria of placentas after delivery, even those from pregnancies ending in caesarean delivery, i.e., free from vaginal contact [197]. This may suggest that the *Lactobacillus* spp. colonizing the placental and vaginal environment play a significant role in the correct course of pregnancy, and perhaps the depletion of this genus in these areas may lead to gestational pathology. 

It has been proposed that gestational vaginal dysbiosis may be a cause of PE [199], and that the nature of the vaginal microbiome may predict the risk of adverse pregnancy outcome [200], and hence should be checked before pregnancy. A nested case–control study found pregestational vaginal dysbiosis including a depletion in the abundance of *Lactobacillus crispatus* to be a risk factor for hypertensive disorder during gestation. Nevertheless, currently, there is insufficient proof that restoration of the vaginal microbiome by vaginal probiotics might protect against preeclampsia. Even so, the use of oral probiotic consumption as a prophylactic against hypertensive pregnancy disorders is still a “hot topic” in research studies. 

Differences have been noted between the gut microbial communities of preeclamptic and normotensive expectant women, and these changes are perceived as an independent risk factor of abnormal placentation and gestational hypertension development [196,201]. Surprisingly, among all the genera of microorganisms typical for the maternal gut, the loss of genus *Lactobacillus* (g_*Lactobacillus*) is strongly linked to the occurrence of the clinical features of preeclampsia. Although this genus is typical of the vaginal environment, and not the intestinal tract, it has been found to colonise the maternal gut during non-complicated gestation; it is believed that this may play an active role in maintaining the correct course of pregnancy. The depletion of *Lactobacillus* appears to be inversely correlated with both systolic and diastolic blood pressure, as well as secretion of urinary protein [201]. This might explain why daily consumption of dairy products supplemented by *Lactobacillus* seems be associated with a lower risk of preeclampsia development, as indicated by a study of nearly 33,400 primiparas [202]. However, the timing of consumption, i.e., probiotic intake, was found to reduce the risk of PE when consumed in late pregnancy and to reduce the risk of preterm delivery in early gestation [196,203]. The initial communications suggesting that probiotic consumption may have a positive impact on pregnancy outcome inspired further clinical trials; however, more time is needed to obtain results: one was recently completed (NCT02693041) whereas NCT05554185 and NCT05436119 are still in the progress.

Similarly, vitamin D has been found to influence the maternal immune system. Studies indicate that low levels of vitamin D disrupt the balance between Th1 and Th2 lymphocyte pattern, favouring the maternal Th1 response. As this Th1/Th2 balance is important for the maintenance of gestation, and the Th1 cytokine profile dominates in preeclamptic pregnancies, vitamin D supplementation during pregnancy appears a good way of preventing PE. This view is also supported by the observation that maternal vitamin D deficiency (<20 ng/mL) increases the risk of occurrence of placental-mediated complications, including preeclampsia, more than fivefold before week 34 of gestation compared to women with a vitamin D level within the normal range. In addition, this risk increased by more than 15-fold when both populations were adjusted for maternal comorbidities diseases such as lupus, diabetes, or a history of adverse pregnancy outcome in the previous gestation [204]. 

This positive influence on preventing PE has been verified numerous times, including a case –control study [205], randomised control trials, e.g., IRCT2017010131695N1 [206], and meta-analysis of randomised clinical trials [207]. Interestingly, the supplementation with both the probiotic and vitamin D does not appear to have any significant effect against preeclampsia, as was observed in a randomised, double-blind, placebo-controlled clinical trial (CT2017060-75623N119) [208]. This divergence might result from the dose of vitamin D, the timing of its initiation and the characteristics of the population enrolled in the studies. 

It has also been proposed that strong antioxidants, e.g., vitamins or polyphenols, may also reduce the risk of preeclampsia. Indeed, disease symptoms are typically preceded by oxidative stress. Reactive oxygen species (ROS) are produced and secreted to the maternal circulation by placental cells as a response to inflammation, hypoxia, and malnutrition, resulting in the presence of high levels of ROS in the maternal bloodstream. These substances disturb the metabolism of cells of the maternal endothelium, thus preventing them from regulating vasoconstriction, and increasing the risk of hypertension. Therefore, it is believed that antioxidants might be beneficial in reducing the risk of preeclampsia. However, numerous studies, including those conducted by the WHO, found treatment with vitamins C and E, two strong antioxidants, to be ineffective in the prevention of preeclampsia development [209,210,211]. Furthermore, a meta-analysis found the use of these vitamins to have negative effects in gestation, being linked with adverse pregnancy outcomes including an increased risk of hypertension among women with a predisposition to preeclampsia [212]. 

The evidence regarding the value of polyphenol supplementation among expectant women to reduce the risk of PE is divided. The polyphenols comprise a heterogeneous group of biologically active compounds of plant origin with antioxidant properties; they are commonly obtained from fruits; vegetables; whole grains; beverages, including coffee tea or wine; as well as dark chocolate and spices [213]. Studies on animal models of preeclampsia indicate that polyphenol consumption is associated with a reduction in blood pressure and proteinuria in pregnant animals [214,215,216,217]. Some clinical trials have found that consuming polyphenols contained in chocolate may reduce the risk of preeclampsia development in pregnant women [218,219]. However, it seems that the source of polyphenols may also have a significant influence on the results of the studies. Indeed, those consumed during persistent tea drinking during gestation increase the risk of pregnancy-induced hypertension and preeclampsia [220,221]. However, the effect of caffeine on gestational blood pressure, a substance commonly present in inter alia coffee, depends on the degree of adaptation by the drinker; while higher caffeine intake increases the risk of gestational hypertension, but only among non-coffee drinkers, regular coffee drinkers experience reduced blood pressure after exposure to daily doses of caffeine [222]. A meta-analysis of clinical trials related to the consumption of various polyphenols by pregnant women does not indicate any association between the intake of antioxidant agents and preeclampsia development [223].

## 6. Medicine of the Future: The Transplantation of Mesenchymal Cells and Their Derivatives for Treatment of Preeclampsia

Numerous animal studies suggest that mesenchymal cells (MSCs) and their derived exosomes may have therapeutic potential in diseases related to the dysregulation of immunological systems, e.g., multiple sclerosis or rheumatoid arthritis [224,225,226,227]. Although this method of treatment has been approved for graft-versus-host and Crohn’s fistula diseases in Japan and Europe, this is not the case in the US [228]. Currently, the FDA has only approved the use of haematopoietic progenitor cells derived from cord blood for the treatment of patients with dysregulated blood production. 

In pregnancy, outside of the decidua, the additional sources of mesenchymal cells are the umbilical cord and the placenta [229]. These placental-derived mesenchymal cells (PDMSCs) emerge from the maternal–foetal interface and retain stem cell-like properties. They exert an immunosuppressive effect on type T lymphocytes, thus calming inflammation, secreting various angiogenesis-promoting factors and presenting antifibrotic activities [230]. The MSCs are also sources of free or exo-encapsulated small molecules, i.e., exosomes rich in proteins and nucleic acids that modulate signalling pathways in nearby cells. Hence, it seems an attractive idea to use the mesenchymal cells or their exosomes as therapeutic agents for preeclampsia treatment. 

Initial studies on animal PE-like models are very promising. Blood pressure, urine protein level, and white blood cell numbers were reduced in preeclamptic rats after injection of the human umbilical cord-derived mesenchymal cells obtained from normotensive women [231]. Moreover, serum from hypertensive animals treated with MSCs demonstrated lower levels of inflammatory cytokines, viz. TNFα and IL-1, in comparison to preeclamptic rats, and these levels were comparable to normotensive dams [231]. 

Similarly, treatment with human placental-derived mesenchymal stromal cells (PDMSCs) appeared to reduce blood pressure and proteinuria in mice with preeclamptic symptoms generated by lipopolysaccharide (LPS) infusion. Additionally, the placentas of preeclamptic mice treated with PDMSCs presented lower levels of sFtl1, IL6, and TNFα in comparison to hypertensive animals. The pregnancies of MSC-treated PE animals ended without foetal reabsorption, and foetal weight did not suggest intrauterine growth restriction [232]. Human mesenchymal stromal cell-derived extracellular vesicles (MEx) were also found to have therapeutic effects on mouse foetuses growing up in the preeclamptic environment [233]. 

While the results obtained from animal studies indicate that MSC therapy might be a candidate for treating preeclamptic women, currently, no anti-preeclamptic therapy based on mesenchymal cells is under examination in clinical trials.

## 7. Conclusions

Currently, no effective pharmacological treatment or methods exist for preventing preeclampsia. The drugs used for the treatment of preeclampsia are generally focused on ameliorating the high blood pressure caused by a sequence of events that began at the beginning of gestation and develop gradually, thus destroying the maternal systems regulating blood pressure. None of these drugs is targeted at the main source or factor responsible for hypertension development, as it remains unknown, similar to the whole pathomechanism of preeclampsia. 

The most common preventive drug, aspirin, recommended by gynaecological and obstetrical societies is a multitasking agent, and may simultaneously block numerous pathways implicated in PE development. Indeed, some reports indicate that 60% of women beginning ASA supplementation before week 16 of gestation do not develop gestational hypertension [21]. However, for some unknown reason, these miraculous results do not occur when treatment is commenced after week 16 of gestation [22,23]. 

**Figure 1:**
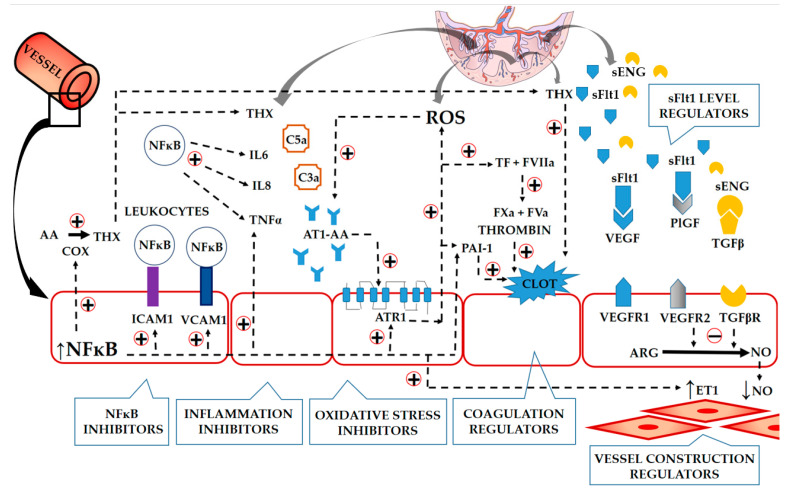
The groups of agents adopted for the prevention and treatment of preeclampsia and their molecular inspirations, i.e.,: NFĸB inhibitors: aspirin, hydroxychloroquine, metformin, proton pump inhibitors, statins, sulfasalazine; inflammation inhibitors: aspirin, eculizumab, metformin, mesenchymal cells and their exosomes, probiotics, statins, sulfasalazine, vitamin D; oxidative stress inhibitors: aspirin, polyphenols, vitamin C, vitamin D, vitamin E; coagulation regulators: aspirin, antithrombin, hydroxychloroquine, mesenchymal cells and their exosomes; sFlt1 level regulators: apheresis, chimera of elastin like-polypeptide with vascular endothelial growth factor type A or type B, small interfering RNA particles; vessel construction regulators: aspirin, hydroxychloroquine, metformin, L-arginine, L-citrulline, proton pump inhibitors, statins, sulfasalazine. Legend: abbreviations: AA—arachidonic acid; ARG—arginine; AT1-AA—angiotensin II type 1 receptor autoantibody; ATR1—angiotensin II receptor type 1; C3a—complement component 3a; C5a—complement component 5a; COX—cyclooxygenase; ET1—endothelin 1; FVa—active coagulation factor Va; FVIIa—active coagulation factor VII; FXa—active coagulation factor Xa; ICAM 1—intracellular adhesion molecule type 1; IL6—interleukin 6; IL8—interleukin 8; NFĸB—nuclear factor kappa B; NO—nitric oxide; PAI-1—plasminogen activator inhibitor 1; PlGF—placenta growth factor; ROS—reactive oxygen species; sENG—soluble endoglin; sFlt1—soluble fms-like tyrosine kinase 1; TF—tissue factor; TGFβ—tumour growth factor beta; THX—thromboxane; TNFα—tumour necrosis factor alpha; VCAM 1—vascular adhesion molecule type 1, VEGFR1—vascular endothelial growth factor receptor type 1; VEGFR2—vascular endothelial growth factor receptor type 2; symbols: 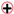 process activation; 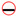 process inhibition; 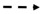 the direction of common relationships between the analysed factors.

Following aspirin, it seems a good strategy to use, in the prevention or treatment of PE, multifunctional drugs controlling cellular pathways that modulate inflammation, oxidative stress, angiogenesis, placental apoptosis, and blood pressure (Figure 1).

It Is known that the pathological processes leading to PE begin in the uterus, where excessive inflammation disturbs the cross-talk between the maternal and foetal systems. These numerous pathways, including those related to NFĸB, might be activated in different orders in maternal and placental cells to generate inflammation; however, they all eventually result in placental insufficiency and maternal endothelium dysfunction, thus giving rise to clinical symptoms. As such, it is desirable for the preventive agents to migrate directly to the uterine space, where they can restore the correct activity of the various intracellular pathways in maternal and placental cells. Additionally, it would be desirable for these drugs to not be transferred through the placental barrier to the bloodstream of the foetus, thus preventing any adverse effects on the foetus. 

Statins, similar to aspirin, are a group of drugs that not only regulate lipid metabolism but also various other intracellular pathways, including those related to inflammation and the production and secretion of placental substances influencing endothelial dysfunction in the mothers [71,77,234]. Numerous studies indicate that these agents are effective in reducing the risk of PE; however, as they also significantly reduce cholesterol level, they also increase the risk of miscarriage or foetal congenital disease [82,83,84,85,88,89]. As such, their general use by expectant mothers is not recommended. 

Statins, as well as other agents whose utility in the process of treatment of preeclampsia has been verified under clinical trials, are well-known medicines approved by agencies such as the FDA for treating various conditions, but this approval does not extend to preeclampsia. The advantage of these drugs is undoubtedly the knowledge about their mechanisms of action on the molecular level. Etanercept, sulfasalazine, and hydroxychloroquine are known for their anti-inflammatory properties, as is eculizumab: a monoclonal antibody acting against complement components. They appear to yield different effects according to their time of administration during gestation; they might decrease the risk of PE development when given at the beginning of gestation, while they might mitigate the inflammation and prolong pregnancy if administered when the symptoms appear; however, due to a lack of knowledge about their influence on the wellbeing of the foetus, the drugs have rarely been tested as preventive agents against preeclampsia in healthy pregnant women who do not require treatment for comorbidities. 

Surprisingly, some drugs may not be fully effective when they are taken by women at high risk of preeclampsia but who are not suffering from the comorbidities for which the drugs are recommended. An example is metformin, an antidiabetic agent that reduces the risk of cardiovascular diseases; while it may also lower the chance of preeclampsia, this is only observed among patients with hyperglycaemia [185]. 

Proton pump inhibitors, recognised by the FDA as safe for expectant mothers, are known to influence various factors implicated in the pathomechanism of preeclampsia. These agents are believed to silence NFĸB activity, and thus inflammation and maternal endothelial dysfunction; they inhibit the ERK pathway and thus apoptosis, and confound various factors disturbing the endothelium or increasing blood pressure by inhibiting the secretion of NO (e.g., sFtl1, sENG, or ADMA) [67,172]. 

Undoubtedly, the most interesting approach to treating preeclampsia is based on the elimination of sFlt1 from maternal circulation. This has been achieved by the use of apheresis devices, which use negatively-charged dextran sulphate to scavenge the sFlt1 factor from the maternal circulation. Although this way of treatment is invasive, it is safe both for the mother and her child and delivers amazing effects: it has prolonged pregnancy for as much as 15 days [145]. 

Another spectacular treatment for PE is based on siRNA technology. In this case, siRNA particles are stabilised by modification and delivered by the use of nanoparticles, e.g., polyamidoamine, to the placental cells. Here, these drugs target the mRNA coding for a soluble fraction of Flt1 but omit the nucleic acid whose product is the membrane, i.e., full version of receptor for VEGF and PlGF factors [149]. This method of treatment is so promising that in 2023, the FDA cleared an investigational new drug (CBP-4888) based on siRNA therapy to treat preeclampsia, as announced by Comanche Biopharma. Similarly, a promising new idea for the treatment of PE is based on the intravenous injection of therapeutic peptides whose original forms had been eliminated from the maternal blood during preeclampsia. These peptides, e.g., VEGF-A or VEGF-B, are stabilised by a nonimmunogenic, engineered carrier that prolongs the lifetime of the therapeutic molecules, thus allowing them for supplementation of maternal bloodstream in VEGF. The VEGF deficiency is the reason for the elevated level of its soluble receptor, i.e., sFlt1, and it is the hallmark of preeclampsia [153]. This mode of action supports the endothelial cells in regulating blood pressure influenced by nitric oxide. Endothelial NO production can also be improved by maternal supplementation with precursors of nitric oxide, i.e., L-arginine and L-citrulline. These amino acids are safe both for the expectant mother and her child and, as indicated by clinical trials, L-arginine effectively reduces the risk of hypertension associated with preeclampsia [161,162,163]. 

Although a number of strategies for the prevention and treatment of preeclampsia are promising in clinical trials, their results remain inconclusive. Hence, any treatment strategy for preeclampsia should be more personalised, as each preeclamptic case might be the result of the activation of different, individual pathological pathways, despite generating common clinical symptoms. Although it may seem obvious, such personalised approaches are presently impossible to realise due to a lack of understanding about the pathomechanism of preeclampsia. Therefore, future research should follow three key directions: (1) identifying new pathways of PE development, individual to the patient, and their diagnostic markers to choose the best method for preventing or treating the disease; (2) creating new substances or methods that can remove the agents provoking the development of PE by the mother; (3) developing medicines that are safe for all expectant women, and which are known to inhibit the pathways responsible for the generation of PE based on individual cases.

## Figures and Tables

**Table 1:** The most common drugs recommended by obstetrical and gynaecological societies for treatment of preeclampsia.

Society	Drug								
Nonsevere hypertension (HA) > 140/90 mmHg									
	Nifedip.	Labet.	Hydral.	mDopa	Oxypren.	Prazos.	Urapid.	nGlic.	Diazo
ACOG	+	+	+						
ESC	+ (IR)	+		+					
FIGO	+	+		+					
ISSHP	+	+	+ II	+	+	+ II			
NICE	+	+		+					
PSH/PCS/PSGO	+	+		+					
SOMANZ	+ (ER) II	+	+ II	+	+	+ II			
Severe hypertension (HA) > 160/100 mmHg or >170/110 mmHg									
ACOG	+ (o)	+ (iv)	+(iv, im)						
ESC	+ (IR, o)	+ (iv)	+ (iv) II	+ (o)					
FIGO	+ (o)	+ (iv, o)	+ (iv)						
ISSHP	+ (IR, o)	+ (iv, o)	+ (iv)	+ (o) II					
NICE	+ (o)	+ (iv, o)	+ (iv)						
PSH/PCS/PSGO	+ (o)	+ (iv, o)	+ (iv)				+ (iv) II		
SOMANZ	+ (o)	+ (iv)	+ (iv)					+ (iv) II	+ (iv)

Legend: Nifedip.—nifedipine; Labet.—labetalol; Hydral.—hydralazine; mDopa—methyldopa; Oxypren.—oxprenolol; Prazos.—prazosin; Urapid.—urapidil; nitroGlic—nitroglycerine; o—oral; iv—intravenous; im—intramuscular; IR—immediate release; ER—extended release; II—second-line treatment drug; ACOG—The American College of Obstetricians and Gynaecologists [30]; ESC—The European Society of Cardiology [31]; FIGO—The International Federation of Gynaecology and Obstetrics [32]; ISSHP—The International Society for the Study of Hypertension in Pregnancy [33]; NICE—National Institute for Health and Care Excellence [34]; PSH/PSC/PSGO—Polish Society of Hypertension, Polish Cardiac Society, and Polish Society of Gynaecologists and Obstetricians [35]; SOMANZ—The Society of Obstetric Medicine of Australia and New Zealand [36].

**Table 2:** Studies examining the influence of pravastatin on preeclampsia.

Study	No. of Recruited Women *	Gest. Age (Weeks)	Statin/ Dose per Day	Observed Results
Constantine et al., 2021 [82] (NCT01717586)	10/10	12–16	Pravastatin (20 mg)	Reduction of PE risk No risk for foetal development
Constantine et al., 2016 [83] (NCT01717586)	10/10	12–16	Pravastatin (10 mg)	Decrease the risk of PE No risk for foetal development
Akbar et al., 2022 [84] (NCT03648970)	87/86	14–20	Pravastatin (20 mg)	Reduction of the risk of preterm PE Reduction of risk preterm delivery Improvement of Apgar score and bright weigh of the baby
Akbar et al., 2021 [85] (NCT03648970)	40/40	14–20	Pravastatin (20 mg)	Reduction of the risk of PE (insignificant) Decrease of risk of preterm delivery Lack of significant differences in the ratio of sFlt1/PlGF before and after pravastatin treatment
Ahemed et al., 2020 [86] (NCT23410175)	30/32 in both groups PE women	24–37	Pravastatin (40 mg)	Insignificant reduction in sFlt1 level in pravastatin group Lack of differences in PlGF and sFlt1/PlGF ratio between placebo and pravastatin group
Brownfood 2015 [77]	4 PE women	20–30	Pravastatin (40 mg)	Stabilisation of clinical features of PE Reduction in sFlt1 after treatment Stabilisation sENG after treatment

Legend: *—No. of recruited women—number of recruited woman for study/control groups; Gest. age (week)—gestational age (week).

## Data Availability

Not applicable.
